# Decellularization and Their Significance for Tissue Regeneration in the Era of 3D Bioprinting

**DOI:** 10.1021/acsomega.3c08930

**Published:** 2024-02-06

**Authors:** Mrunmayi Gadre, Meghana Kasturi, Prachi Agarwal, Kirthanashri S. Vasanthan

**Affiliations:** † Manipal Centre for Biotherapeutics Research, Manipal Academy of Higher Education, Manipal 576104, Karnataka, India; ‡ Department of Mechanical Engineering, University of Michigan, Dearborn, Michigan 48128, United States

## Abstract

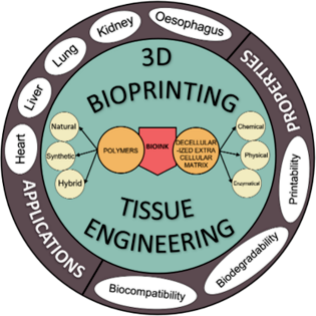

Three-dimensional bioprinting is an emerging technology that has high potential application in tissue engineering and regenerative medicine. Increasing advancement and improvement in the decellularization process have led to an increase in the demand for using a decellularized extracellular matrix (dECM) to fabricate tissue engineered products. Decellularization is the process of retaining the extracellular matrix (ECM) while the cellular components are completely removed to harvest the ECM for the regeneration of various tissues and across different sources. Post decellularization of tissues and organs, they act as natural biomaterials to provide the biochemical and structural support to establish cell communication. Selection of an effective method for decellularization is crucial, and various factors like tissue density, geometric organization, and ECM composition affect the regenerative potential which has an impact on the end product. The dECM is a versatile material which is added as an important ingredient to formulate the bioink component for constructing tissue and organs for various significant studies. Bioink consisting of dECM from various sources is used to generate tissue-specific bioink that is unique and to mimic different biometric microenvironments. At present, there are many different techniques applied for decellularization, and the process is not standardized and regulated due to broad application. This review aims to provide an overview of different decellularization procedures, and we also emphasize the different dECM-derived bioinks present in the current global market and the major clinical outcomes. We have also highlighted an overview of benefits and limitations of different decellularization methods and various characteristic validations of decellularization and dECM-derived bioinks.

## 1.0 Introduction

Tissue engineering is an interdisciplinary field which includes biology, medicine, and engineering. This technique aids in the fabrication of *in vitro* tissues or organs of interest by creating biomimetic scaffolds that mimic the native microenvironment of the respective tissue or organ for regeneration/replacement. This will address the challenges associated with organ transplantations that occur due to a lack in the availability of healthy donors, immune rejections, etc. The 3D printing technique helps in the process of fabricating functional and complex yet precise tissue constructs by depositing the bioink intricately in a layer-by-layer manner for spatial arrangement of cells in all of the layers that will eventually promote tissue formation. The bioprinted scaffolds help in the adherence of cells and establish cell signaling, thereby resembling the native tissue. To achieve the fabrication of relevant functional frameworks, it is critical for the scaffold to mimic the mechanical stability and achieve appropriate strength similar to the native tissue, which can be achieved by selection of appropriate biomaterial. Additionally, to enhance the cell attachment and proliferation leading to tissue regeneration, biochemical cues are required, which can be supplied by the components of biomaterials. Research over the last few decades reported that the use of natural biomaterials for the fabrication of artificial tissues as a better fit, owing to the ability to mimic the tissue microenvironment as they are biocompatible, biodegradable, and noncytotoxic, promotes cell attachment, proliferation, differentiation, and cell signaling.^1^

The extracellular matrix (ECM) is a 3D network comprising an array of macromolecules organized in a specific manner to form a stable structure that contributes to the mechanical properties of the tissue. The ECM is also a reservoir of proteins, growth factors, and bioactive molecules that are organized in a specific manner, having functional importance for fundamental cell behaviors such as adhesion, migration, proliferation, differentiation, and apoptosis, and supports biomechanical properties.^2^ The ECM biomolecules are specific to each tissue, and thus scaffolds mimicking the ECM properties have more physiological relevance. Recent studies showed that the use of dECM as a material or a scaffold additive enhanced the physiological relevance of the tissues for development and regeneration.

The decellularization process to obtain dECM can be through physical, chemical, and enzymatic methods. Physical methods of decellularization include freeze–thaw cycles, high hydrostatic pressure, or supercritical carbon dioxide (scCO_2_). Chemical methods use surfactants to solubilize cell membranes and dissociate their internal structure, while enzymatic methods employ the use of enzymes such as trypsin, Dispase, phospholipase A2, etc.^3^ After successful decellularization, several characterizations are performed on the dECM tissues to test for the efficiency of the process in order to avoid immunogenic rejection for *in vivo* application. Tests are performed to ensure the preservation of the ECM structure and to access the biological and mechanical properties of the scaffold. They include validating the DNA content and protein content to quantitatively assess the presence of nuclear and ECM component content; visual characterization includes staining procedures to observe the presence/absence of nuclear and ECM components. The cyto and immune compatibility is tested by measuring the cell proliferation and cell viability via indirect and direct methods to validate the dECM interaction with the cells.^4^ To assess the mechanical rigidity, tensile and compression analysis are performed with reference to the native tissue.^5^

The application of dECM for wound healing,^6^ implantation fillings,^7^ reconstruction procedures,^8^ and disease modeling has been widely reported. Decellularized tissue-based products like the Avance Nerve Graft,^9^ AlloDerm Regenerative Tissue Matrix,^10^ Oasis Wound Matrix,^11^ etc. are being used in clinics for regenerative purposes. *In vivo* research has proven that a 3D-printed skin patch comprised of endothelial progenitor cells laden with adipose-derived stem cells accelerates wound closure, re-epithelization, and neovascularization as well as blood flow.^12^ Another study involving the use of a myocardial matrix has proven an increase of endogenous cardiomyocytes in the infarct area and improved cardiac function.^13^ Despite many reports on using dECM as a scaffold, the research is in progress and requires optimization and a standardized protocol for establishing the decellularization techniques specific to each organ.

## 2.0 Decellularization

**Figure 1:**
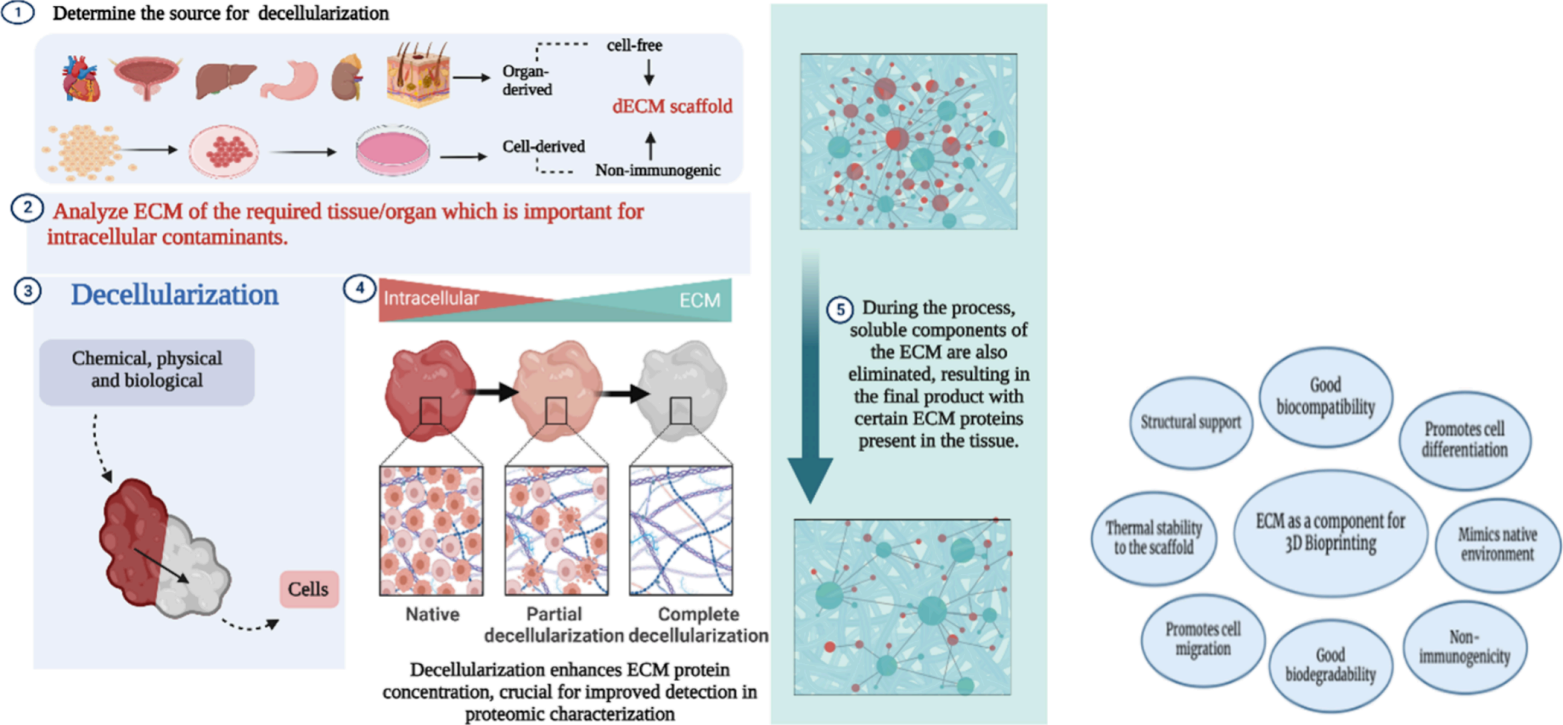
Overview of the decellularization technique and various applications of the dECM component for *in vitro* studies.

The process of decellularization is the removal of all cellular and nuclear components from the tissue or organ to retain the native architecture and to preserve biochemical and biomechanical components of the ECM.^14^ The dECM is rich in various proteins which include collagen, glycosaminoglycans, elastin, microfibrils, proteoglycans, and a wide range of growth factors. The main application of this ECM structure is to create a biocompatible microenvironment for the cells to attach, function, and proliferate.^15^ There exist multiple techniques of decellularization, which include physical (temperature, force, pressure), chemical (detergents, solvents, acids), and biological (enzymes) treatments. Each treatment is unique and produces dECM and requires optimization based on application. The structure of dECM is highly specific to the tissue source, and this helps with remodeling the tissue function as per the requirement.^16−18^ Decellularization ultimately aims to deliver dECM that can be used as scaffold material or the decellularized organs that retain the topography and dimension, which can be seeded and used. Post successful decellularization, the process also requires the purification of the contaminants and removal of residual agents that were employed. Figure 1 provides an overview of decellularization and the application of dECM as a component. The entire process can be subdivided into three steps including washing, rinsing, and sterilization steps. The washing is the preliminary step which involves the use of different agents to lyse the cells and break down the tissue,^16^ which is followed by rinsing to remove the detergents that are cytotoxic and can inhibit the cellular proliferation.^19^ Finally, via the sterilization step, antigenic and microbial components are removed to reduce the immunogenic responses in dECM.^20^ The primary challenge is the removal of the cellular components due to their sticky and adherent nature toward the ECM proteins, but complete removal of cellular materials can be achieved while retaining the proteins if optimized protocols are followed. Due to the increased demand for scaffold fabrication technology, which involves dECM for producing surgical mesh for routine use in clinics, dECM is derived from various allogenic or xenogeneic sources. Researchers have suggested the use of a tissue-specific source for isolation of the ECM for developing the cell functions. Widely studied tissue sources for obtaining the dECM include the liver,^22−24^ respiratory tract,^25,26^ nerve,^27−29^ adipose^30^ and mammary gland,^31^ heart valves,^32−38^ blood vessels,^39−42^ skin,^43^ skeletal muscle,^44^ tendons,^45^ ligaments,^46^ small intestinal submucosa,^47−49^ and urinary bladder.^50−52^ Various characteristics have been proven in the use of the ECM including the influence on cell mitogenesis and chemotaxis,^53,54^ cell differentiation,^55−60^ and inducing positive host remodeling responses.^61−63^ Immunological rejections and inflammatory responses by the host take place due to the cellular antigens present in the xenogeneic and allogenic sources; however, during the decellularization process these factors are washed out during the process.^64−67^ The absence of HLA antigen expression in the dECM proves their biocompatibility. The immune-conducive environment and cytocompatibility can be proved by various characterizations. The use of detergents and the physical distortions have proven to increase the biocompatibility of the dECM. The most commonly used process for decellularization involves the combination of chemical and physical agents wherein the physical agents alone have been proven to be insufficient for decellularization, and hence there is a need to be combined with chemical agents.

### 2.1 Chemical Treatment

#### 2.1.1 Acids and Bases

Acids and bases used for decellularization will solubilize the cytoplasmic component of the cell and remove nucleic acids. Widely used acids are acetic acid,^68^ peracetic acid (PAA),^69,70^ hydrochloric acid, and sulfuric acid,^71−75^ while the bases including calcium hydroxide, sodium sulfide, sodium hydroxide, and ammonium hydroxide are usually known for their harshness and are usually applied for removing the hair and dermal samples during the early stages of decellularization. However, these agents are also known for complete elimination of growth factors in the ECM and significantly reduce the mechanical strength of dECM when compared to other agents.^76^

#### 2.1.2 Hypotonic and Hypertonic Solutions

Hypertonic and hypotonic solutions are known to rinse out cellular residues from tissue following their lysis. The hypertonic saline dissociates the DNA content from proteins,^77^ and hypotonic solutions cause immediate cell lysis through simple osmotic effects.^40^ Various studies have reported the use of alternative immersion of the hypertonic and hypotonic solutions to obtain maximum effect.^78−81^ Widely used hypertonic solutions include sodium chloride, and hypotonic solutions include Tris-HCl. Since they are known for incomplete removal of the cellular remains in the dECM, additional treatment with chemicals or enzymes has yielded the desired result.

#### 2.1.3 Detergents

Ionic, nonionic, and zwitterionic detergents are generally used in the decellularization as their treatment will cause the solubilization of cellular membranes and promotes dissociation of DNA from proteins, aiding in the effective decellularization.^82,83^ However, few of these agents tend to dissociate and disrupt ECM proteins which has been confirmed via proteomics studies.^84,85^ The removal of proteins in ECM is directly proportional to the amount of time the ECM is exposed to the detergents^43,86^ and the involvement of multiple agents. Widely used ionic detergents include sodium dodecyl sulfate (SDS), sodium deoxycholate, and Triton X-200, whereas the nonionic agent includes Triton X-100 and zwitterionic agents like 3-[(3-cholamidopropyl)dimethylammonio]-1-propanesulfonate (CHAPS) and sulfobetaine-10 and sulfobetaine-16. Nonionic agents are relatively mild when compared to other agents as they disrupt the lipid–lipid and lipid–protein interactions.^87^

#### 2.1.4 Alcohols

Glycerol, isopropanol, ethanol, and methanol cause the dehydration of the cells, resulting in cell lysis.^75^ They are more effective than lipase as an agent as they remove the lipids from tissues,^30,88^ while these are also known to precipitate proteins^89^ and damage the ultrastructure of the ECM.^90−92^ Hence, the use of the alcohol as a decellularizing agent is not preferred over the other agents.

#### 2.1.5 Other Dolvents

Acetone and tributyl phosphate (TBP) act directly by removing the lipids from the tissues.^93−95^ However, they are also known for their application in the fixation of tissues, and when used as decellularization agents they result in damage to the ECM.^96,97^ They also act as cross-linkers for the ECM and result in increased stiffness as they find application as a chaotropic agent in decellularizing tendon and ligament grafts.

### 2.2 Biologic Treatment

#### 2.2.1 Enzymes

The enzymatic agents for decellularization include nucleases, trypsin, collagenase, lipase, dispase, thermolysin, and α-galactosidase which aid in the removal of the residual cellular components. The major drawback of this method is the failure to completely decellularize and the demand for additional use of other agents. Nucleases like DNase and RNase aim to cleave the nucleic acid sequences and lyse the cells in the tissues.^25,98,99^ Endonucleases such as benzonase are more effective, as they act on cleaving the nucleotide sequences. Trypsin is a serine protease, which is an effective agent but also effects the ECM protein by cleaving collagen and elastin and shows better prevention of glycosaminoglycan content.^35,100−102^

#### 2.2.2 Nonenzymatic Agents

Nonenzymatic agents like EDTA and EGTA act as a chelating factor by forming a ring around the central metal ion to bind and isolate the nuclear content (Ca^2+^ and Mg^2+^).^103,104^ It is very likely that these agents can subtly disrupt the protein–protein interactions through their mode of action.^105^ The chelating agents have been unsuccessful in complete decellularization even after applying additional agitation, and hence the use of additional agents like detergents and enzymes is necessary to obtain the desired result.^106−109^ Latrunculin is another nonenzymatic agent which is naturally cytotoxic and acts as a toxin and is also applied in decellularization.^110^

### 2.3 Physical Treatment

#### 2.3.1 Temperature

Temperature can also play an important role during decellularization, and hence, it has been largely combined with chemical agents as the freeze–thaw cycle can effectively aid in easy lyses of cells. One cycle of the freeze–thaw cycle can effectively aid in removing the leukocyte infiltration.^111^ Though multiple cycles of freeze–thawing can also be applied for decellularization, there is a requirement of another chemical agent for successful decellularization.^30,57,87,112,113^ Few studies have reported minute disruption of the ECM ultrastructure, and hence the application of this method is limited.^75,114^

#### 2.3.2 Force and Pressure

Under certain forces and pressures applied to cells, they tend to burst, which is used for decellularization. This method has to be applied in combination with additional chemical treatment for the complete dissociation of the cells from the tissues. A protocol involving the application of hydrostatic pressure may require relatively lesser time when compared to the detergents and enzymes.^105,113^ Due to ice crystal formation, it may lead to destruction of the ECM structure as it is associated with the rise in entropy.^114^

#### 2.3.3 Nonthermal

Another popular protocol is the nonthermal irreversible electroporation (NTIRE) which is applied for decellularization. In this technique, electrical pulses are passed throughout the tissue for certain durations which results in the microspore formation in the cell membranes, promoting loss of cellular homeostasis, eventually leading to cell death.^115,116^ As this is a comparatively new protocol, further standardization is required, and certain studies showed ECM components are retained by maintaining appropriate heat during the process.^117^

**Figure 2:**
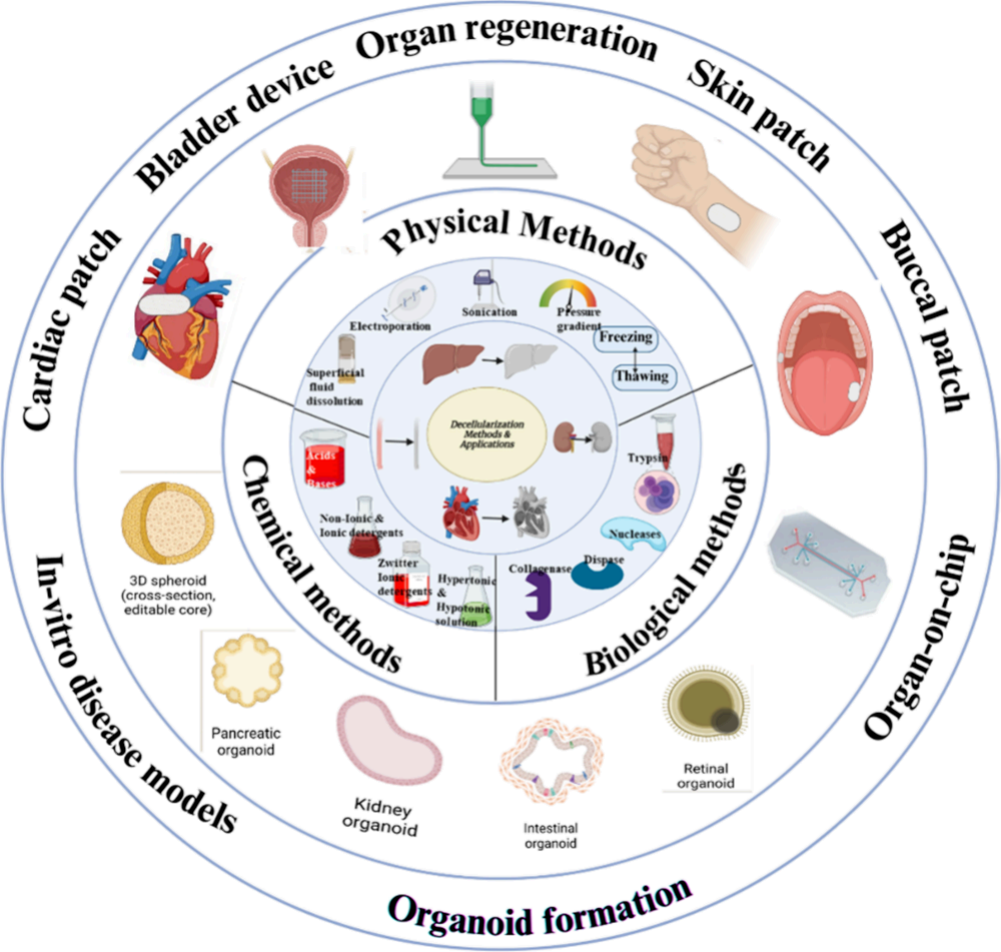
Various decellularization techniques and applications of the derived dECM. Photo credits: Biorender.

**Figure 3:**
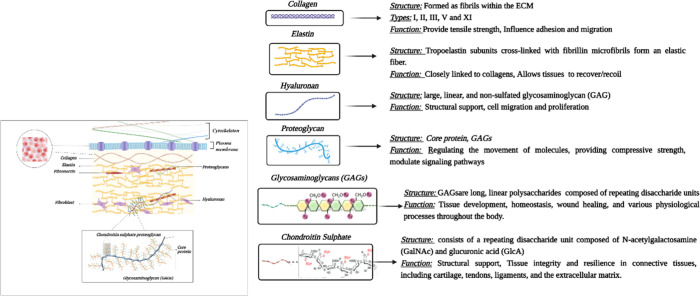
Varied roles of ECM proteins.

Table 1 describes different types of decellularization applied to various organs and the types of decellularizing agents applied in the process. The end product of decellularization is to obtain proteins that are present in the ECM. An overview of the different decellularization techniques shown in Figure 2 and Figure 3 provides a brief structure of the ECM and the importance of the ECM proteins. These proteins present in the dECM help to provide significantly higher cellular viability and functionality.

**Table 1:** Summary of Different Decellularization Agents and Their Impact on the ECM^a^

Decellularization agents		Action by agents	Influence of the agents on the ECM and cells	Organs	References
Chemical agents	Acid	Acetic acid, PAA^1^, HCl^2^, H_2_SO_4_^3^	Removal of DNA by solubilizing the cellular contents and disrupting the nucleic acids, it can also lead to denaturation of ECM and reducing their strength.	Urinary bladder, small intestinal submucosa, stomach submucosa, thoracic aortic aneurysms	(118−121)
	Basic	Calcium hydroxide, sodium sulfide, and sodium hydroxide and ammonium hydroxide	Removal of DNA by Denaturing plasmid and chromosomal DNA it can also lead to collagen damage and reduction in the growth factor and also hamper mechanical property of ECM	Bladder submucosa, corpora, urinary bladder	(122−125)
	Hypotonic and hypertonic solutions	NaCl^4^	It causes shock by osmosis and is generally less efficient in removal of DNA and there is less disruption in the ECM	Intestine, umbilical cord, carotid arteries	(126−129)
	Detergents	SDS^5^ Triton X – 200 Triton X – 100 CHAPS^6^, SB-10^7^	The most efficient method for decellularization and helps in removal of all the cellular content by solubilizing the membrane of cell and they may cause disruption in the ECM ultrastructure.	Nerve tissues, ovary, liver, lung, aorta, esophagus	(50, 130−133)
	Alcohol	Ethanol	Cell lysis by dehydrating the tissue May disrupt ECM ultrastructure Disrupts protein–protein connections and enhance collagen cross-linking	Lamina propria, temporomandibular joint disk, heart	(70−73)
	Organic solvents	Acetone, TnBP	Lysing of the cells by dehydration leads to removal of genetic materials. It also causes the cross-linking of ECM content leading to hardening of matrix.	Pericardium, diaphragm	(134, 135,136)
Biological agents	Enzymes	Trypsin, DNase^8^, RNase^9^	They lead to the cleavage of cells by detaching the cells from the adhered proteins. Longer exposure can lead to damage of ECM.	Cornea, limbal stroma, skin, peripheral nerve	(39, 40, 137)
	Nonenzymes	EDTA^10^, EGTA^11^	They work by disruption of adhered cells and usually combined with other agents due to their low efficiency.	Heart, kidney	(138−140)
Physical agents	Temperature	Freeze–thaw	This method leads to the crystallization of water hence causing cell death, which may also disrupt the ECM.	Tendon, cornea	(141, 142)
	Force and pressure	Hydrostatic pressure, agitation	Cell death may take place but complete removal takes place with aid of other agents. It may also cause damage in the biomechanical properties of ECM.	Aorta, blood vessels	(94, 143, 144)

a PAA^1^ - Peracetic acid, HCl^2^ - Hydrochloride acid, H_2_SO_4_^3^ - Sulfuric acid, NaCl^4^ - Sodium chloride, SDS^5^ - Sodium dodecyl sulfate, CHAPS^6^ - 3-[(3-Cholamidopropyl)dimethylammonio]-1-propanesulfonate, SB-10^7^ - sulfobetaine 10, DNase^8^ - Deoxyribonuclease, RNase^9^ - Ribonucleases, EDTA^10^ - Ethylenediaminetetraacetic acid, EGTA^11^ - Ethylene glycol tetraacetic acid.

## 3.0 Characteristic Evaluation for Decellularization

Characteristic evaluation of the dECM is performed to validate the amount of residual cellular components that is retained post decellularization. There exist various standard evaluations for characterization which are performed on the dECM to validate the amount of residual cellular components that remain post decellularization. These evaluations play a critical role in deciphering the *in vitro* and *in vivo* cytocompatibility.^145−147^ The quantification of cell components, including the double-stranded DNA, mitochondria, or molecules associated with membranes such as phospholipids, is validated in the dECM. There is a threshold of residual cellular components that is sufficient for a negative response in the host that might lead to remodeling. These responses depend on various factors like the source and the site of dECM implantation and the host immune function.^37,75^ There are various protocol reports to determine the efficacy of decellularization, but they lack well-defined quantitative matrices. Based on the results from various studies where a successful constructive *in vivo* remodeling has been established and adverse negative host responses have been observed, a few minimal criteria meet the requirements to satisfy the evaluation of decellularization. The primary focus is on the amount of residual DNA which is directly proportional to the intensity of host reactions as it is ubiquitously present throughout the tissues.^148^ A comparative protocol for evaluating the dECM is required as it is obtained from native tissues. One such protocol includes dsDNA per mg of ECM dry weight which must be <50 ng^149^ and second <200 bp DNA fragment length^150^ and has a lack of visible nuclear material in tissue sections stained with 4′,6-diamidino-2-phenylindole (DAPI) or Haematoxylin and Eosin (H&E).^82^ The first and second criteria can be quantified easily by various isolation protocols along with gel electrophoresis, and the third criteria for evaluation is through histological staining and immunofluorescence for inspection to determine the presence of nuclear components. There are alternative stains including the Masson’s trichome, Movat’s pentachrome, or Safrin O Alcian Blue staining (collagen) and Verhoeff Van Gieson (elastin), periodic acid Schiff (sugars), and Oil Red O staining (lipids) that have been applied to evaluate the protein components of the dECM.^151^ Immunohistochemical staining using actin and vimentin, DAPI, and hoescht is performed^152^ to evaluate the presence of DNA in dECM.^153−155^ Scanning electron microscopy (SEM) and transmission electron microscopy (TEM) are used to assess the microstructural integrity of the dECM.^156^ Proteomics and Fourier infrared spectroscopy (FTIR) analysis were used to assess the proteins and cytokines present in the dECM. A mandatory investigation involves the evaluation of dECM for the immunologic rejections via qualitative real-time polymerase chain reaction (qRT-PCR) that determines the nanogram quantities of ECM proteins. The relative gene expression ratio assesses the immune cell responses, where the tissue after harvesting is homogenized and run through the RNeasy kit and compared with the decellularized samples for the gene expressions.^157^ Surface marker analysis for determining the HLA-DR is another technique to examine and ensure the compatibility of the dECM for the recipient is performed. As the techniques further evolve and new protocols for decellularization are discovered, these above criteria for evaluation of decellularization also modify or at the most are supplemented with other protocols to maintain the consistency and the quality for assessment. It is not only crucial to evaluate the dECM samples for the absence of the cellular components but also important to confirm the presence of the desired ECM components that are the proteins that are involved in adhesion. The above-mentioned procedures for assessing the dECM quality mainly include the removal of cellular components and the retention of the extracellular matrix components.

The gelation of bioink plays a crucial role in bioprinting 3D scaffolds. The bioinks have direct contact with cells to provide structural support and also decide the chemical and physical properties of bioinks.^158^ Ideally, bioinks used during bioprinting must be well characterized for their physicochemical properties. Rheology is the study of flow properties of materials under external forces.^159^ Unfortunately, most of the data obtained from rheological tests lack the contextual relationship of the rheology to the 3D bioprinting results. Recent studies must validate the correlation between the rheology of bioinks and their shape fidelity. Rheological properties of bioinks also greatly influence printing fidelity and cell durability in 3D bioprinted scaffolds.^160^ There are various rheological characterizations such as storage modulus (G¢), loss modulus (G†), and viscosity (*Z*) to predict the potential of the bioink for 3D bioprinting using specific displacement or force like oscillation (back and forth) or rotation (unidirectional).^161^ Storage modulus is the measure of elastic energy, while loss modulus is the measure of the viscous portion or dissipated energy within the bioink.^162^ Oscillatory measurements are implied in the calculation of both the storage and the loss moduli. Rotational tests also aid in measuring the viscosity and calculate the resistance in the flow of the material.^163^ Another important rheological property of bioinks is the viscosity of bioink, which is the ability of the bioink to flow through the reservoir and needle onto the printing surface during 3D bioprinting. The higher viscosity enhances the stability and longer durability of the 3D bioprinted structure and cell viability, while a decrease in viscosity hinders printability. Increased viscosity can lead to blockages at the nozzle tip, which require additional adjustments according to the nozzle tip size. In bioink formulations, viscosity control is achievable through the regulation of factors like molecular weight, polymer concentration, the number of additives, temperature, and pre-cross-linking.^164^ Generally, oscillatory amplitude or frequency sweep is used to determine the storage and loss moduli and a rotational shear-rate sweep to determine viscosity for characterizing bioink.^165^ As a measurement of bioink performance, storage and loss moduli are determined in pre-cross-linked or post-cross-linked bioinks to understand the difference in the performance of bioinks. After extrusion, bioink must get cross-linked to have a stable structure. These rheological characteristics are crucial to define the printability of bioink. Other major rheological properties affecting the final characteristics of the 3D bioprinted tissues include flow behavior, viscosity, shear stress, and viscoelasticity.^166^ Flow behavior can be categorized as Newtonian and non-Newtonian to indicate bioink resistance to shear deformation, shear stress (or viscosity), and shear rate.^167^ Bioinks containing dECM display non-Newtonian flow and shear-thinning characteristics, facilitating the smooth flow of bioink without causing nozzle blockages in 3D bioprinters.^168,169^ Bioink viscosity plays a crucial role in determining the shear stress experienced during bioprinting procedures, potentially impacting cell survival and proliferation. Elevated shear stress levels can pose a risk of causing damage to cells.^170^ Therefore, dECM-based bioinks are favored when they exhibit low shear stress rates at moderate pressures, as this enables optimal printing precision and the preservation of live cells in both *in vitro* and *in vivo* environments.^171,172^ The viscoelastic properties of dECM-based bioinks are determined through dynamic measurements of storage and loss moduli, which vary with shear stress, strain, frequency, or time. The storage modulus, or elastic modulus (*G*′), represents the energy stored within the material and is recoverable during each deformation cycle. Conversely, the loss modulus, or viscosity modulus (*G*″), indicates the energy lost as viscous dissipation per deformation cycle. Consequently, in 3D bioprinting, *G*′ and *G*′′ are linked to elastic shape retention and viscous flow, respectively.^173,174^ The viscoelastic properties are significantly influenced by the type of bioink, its concentration, and the applied cross-linking, playing a crucial role in interactions between cells and bioink as well as in the porosity and degradation of 3D bioprinted structures. Additionally, viscoelasticity governs the structural stability and integrity of the bioink, influencing cell proliferation and differentiation.^175^ The damping factor, also referred to as tan(δ) (equal to *G*′′/*G*′) or loss tangent, serves as a valuable indicator of the relationship between the viscous and elastic deformational properties. An optimal dECM-based bioink should strike a balance between the structural integrity of the bioink and the uniformity of bioprinting when the damping factor falls within the range of 0.2 to 0.6. However, nozzle blockage occurs when tan(δ) is below 0.2, while poor shape retention is observed when it exceeds 0.6.^176^

Smoak et al. characterized the fabricated electrospun scaffolds derived from decellularized muscle. Their study focused on tailoring the scaffolds tunable in the aspect of physicochemical properties and at the same time retaining the matrix components’ pro-regenerative property. For decellularization, the chemical method using Triton X-100 and hypotonic and hypertonic salt solutions was employed. This resulted in highly porous scaffolds, allowing for the easy facilitation of nutrients and metabolic waste transportation. Employing electrospinning has provided them a platform to generate desired fiber orientation, change mechanical properties, and improve the degradation kinetics, which provided good cell attachment, migration, and differentiation. For the biochemical characterization of dECM, the group carried out estimation of total DNA, protein, collagen, and sGAG and compared them to the contents in the native. Images by confocal microscopy were captured to measure the swelling in the dry and swollen dECM scaffolds, which was measured by the change in the thickness in the diameter upon contact with phosphate buffered saline. The porosity of the electrospun scaffolds including different groups was calculated by using the confocal *Z*-stacks which were then calculated by using the software ImageJ. The white pixel area (Afiber) and black pixel area (Atotal) were used and calculated by [1 – (Afiber/Atotal)] which was known as epsilon. Mechanical properties of the scaffolds were measured using a mechanical testing machine to measure the tensile modulus in different groups. This group claims that they have developed a novel protocol to develop the electrospun muscle dECM scaffolds without the need of a carrier polymer.^177^

Boso et al. developed a decellularized porcine diaphragm hydrogel for mimicking the complex skeletal muscle extracellular matrix. The hydrogel properties including being biochemical and biocompatible were characterized for adapting *in vivo* applications using the chemical and enzymatical method of decellularization, which involves 4% sodium deoxycholate, DNase-I, and sodium chloride and genipin as a cross-linker. The hydrogel coloration was directly proportional to the amount of cross-linking in the hydrogel and characterized by scanning electron microscopy, and the pore sizes were measured and calculated using ImageJ. Fluorescence recovery after photobleaching was carried out to determine the kinetics of diffusion after hydrogel formation followed by immunofluorescence analysis where the presence of any antigens in the hydrogel was analyzed. Total collagen and hyaluronic acid were estimated by kit-based assays followed by the enzymatic degradation study, where the scaffolds were subjected to collagenase I and monitored over time to evaluate the rate of degradation. Rheological analysis was performed to measure the storage and the loss modulus followed by the turbidimetric gelation kinetics where the gelation rate, half gelation time, and lag time were measured. The study claims to demonstrate that the decellularized porcine diaphragm hydrogels can be represented as useful biological products for repairing the defected diaphragmatic muscle when used as a relevant acellular patch alone.^178^

## 4.0 Benefits and Limitations of Decellularization

As there is an increased need and demand for organ transplantations which often leads to host immune rejections, finding an alternative solution to overcome the issues remains the top priority in the health care section. Commercially available dECM products for repairing soft tissues, such as dECM as 2D coatings (TCPS coating)^179^ and hydrogels,^180^ are widely evaluated as alternatives. The dECM-based bioinks have been widely used as additive biomaterials in 3D bioprinting for fabricating functional organs^21^ that can enhance the native structural similarity and composition of the scaffold. Furthermore, products fabricated from the bioinks are applied in studies for discovering their potency and ability for cellular differentiation.^181^ The hydrogels consisting of dECM play a crucial role in clinical platforms due to their thermo-reversible properties.^182^ The dECM hydrogels predominately involving various tissue sources such as cardiac,^183,184^ adipose,^185,186^ tendon,^187−189^ skeletal muscle,^190−192^ bone,^193^ cartilage,^194,195^ meniscus,^196,197^ dermis,^198,199^ spinal cord,^200−203^ brain,^204^ pancreas,^205^ lung,^206,207^ liver,^208−210^ and umbilical cord^211^ are available. Drug screening studies, drug delivery vehicles, and regenerative tools are the major applications of dECM hydrogels.

Though there has been extensive and continuous research to improve the decellularization techniques, there are few limitations to the application of the products derived from decellularization that include lack of standardized decellularization protocols and evaluation techniques post decellularization. These include no standard protocols for each organ, lack of systematic evaluation to avoid immune responses, and rejections from the xenogeneic source.^212^ Although there are a wide variety of sources available to derive dECM, including the allogeneic and xenogeneic background, there are likely to be high immune rejections by the host body if not completely decellularized. In addition to this, there is a necessity to understand various factors like the composition, properties, and structural characteristics of dECM derived from various sources. Establishing a standard sterilization protocol for dECM-derived products will have a positive impact on recipient tissues.^213^

## 5.0 Bioinks

**Figure 4:**
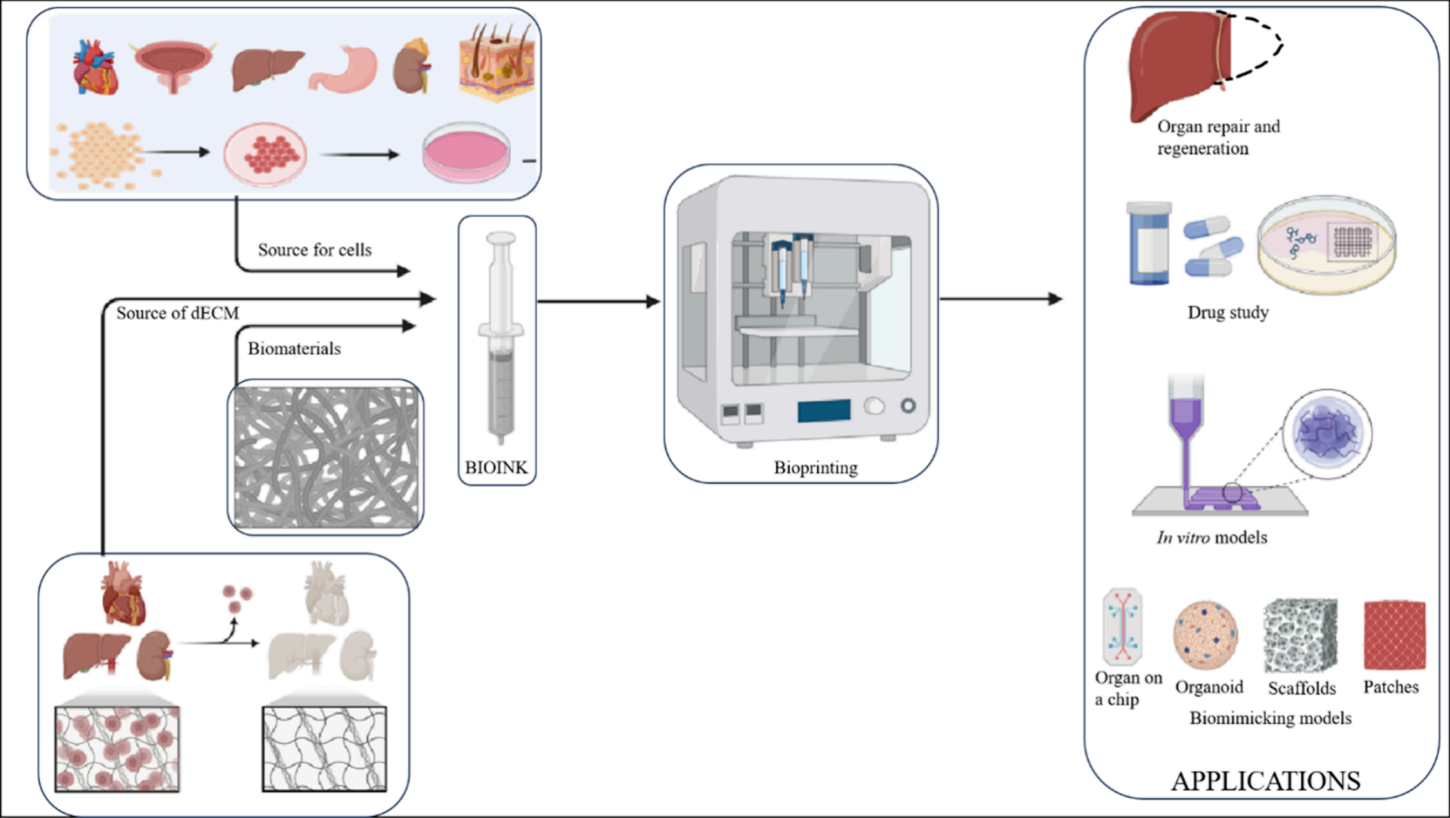
Generalized process of generating *in vitro* models using dECM-derived bioinks.

Bioinks are formulated for *in vitro* 3D bioprinting to develop 3D constructs for multiple biomedical applications. Bioinks comprise biomaterials (natural or synthetic polymers), dECM, and live cells which are printable, biocompatible, mechanically stable, and biodegradable. 3D bioprinting is considered as a ground breaking tool in the field of regenerative medicine and tissue engineering. The dECM-based bioinks can be formulated using sterilized decellularized materials and polymeric biomaterial with an appropriate shear thinning phenomenon to obtain printability. The biophysical properties of 3D bioprinted constructs such as the porosity and mechanical strength are based on the constitution of the bioink.^213^ The generalized process of generating dECM-derived bioinks is briefly outlined in Figure 4. The optimization of concentration and ratio of all the components in bioink also determines the stability of the 3D bioprinted construct. Multiple factors are considered during the synthesis of bioink (the processing of biomaterial, gelation of the bioink, source of the dECM, solubility, and optimal pH). Physical cross-linkers like light and heat, chemical cross-linkers like carbodiimide, microbial transglutaminase, and glutaraldehyde, and photo cross-linking with UV or visible light are done post printing to obtain stable 3D bioprinted constructs.^214^

### 5.1 Properties of dECM-Derived Bioinks

The dECM is created by the decellularized biological components to form a printable ink with the purpose of preserving the components present in the ECM. There are few parameters which determine the quality of the bioink such as printability, biocompatibility, mechanical properties, and *in vitro* degradability, and these properties might vary depending upon the target application. Printability is an important factor for fabricating a 3D bioprinted scaffold as it is important for a bioink to maintain its rheological properties that will eventually maintain the shape.^215^ Processing parameters that can affect the printability of bioink include the composition, scaffold design, and printing technology applied.^216^ The bioprinting techniques such as inkjet printers have limitations in the viscosity of the bioink, while the microextrusion printers require the bioink to have certain specific cross-linking capacity. Biocompatibility results from integration of 3D bioprinted product with the cells without triggering any immune response as they are efficiently able to interact with the host microenvironment, provide suitable conditions for cell growth, and promote cellular adhesion and proliferation along with the expression of cell-specific functions.^217−219^ The presence of antigenic components in the bioink leads to high inflammatory responses, and hence selection of appropriate components in the bioinks is essential.^220^ A combination of the bioink with bioactive cues leads to an increase in the biocompatibility of 3D bioprinted constructs when implanted in the host.^221^ Mechanical stability is the strength to retain the 3D bioprinted structure and protect the cells loaded in bioinks from collapsing. The structure must be able to resist or produce certain forces for mechanical leverage for longer duration for the construct to function for a longer duration. The mechanical strength exerted by the 3D bioprinted structures is to be aligned with the strength of the native tissue. The main component of the bioink that contributes to stability is the use of cross-linking agents that help the other components maintain the structure in the 3D bioprinted scaffolds. In 3D bioprinting, the biophysical properties of bioprinted structures must match the host microenvironment; hence, use of organ-specific dECM is an important component of bioink, and the role of synthetic polymers adds to improving the mechanical properties. The mechanical stability of the bioink is determined by the reaction and the duration of the components of the bioink in the implant microenvironment. Another factor which affects the mechanical stability is the appropriate size of the pores in bioprinted constructs, for example, the bone tissue grafts.^222^ One of the studies suggests that bovine-derived cancellous are acknowledged as the closest with strong mechanical properties as a xenograft to human bone for regeneration, and the autografts are also considered safe products which have been used in regular clinical practices for bone reconstructive surgeries.^223^ Biodegradability is the ability of the materials in the bioink to break down into simpler components when exposed to various factors. Gradually as the time passes, the degrading starts as the cells start to proliferate and migrate, which integrates with the ECM in the construct. There is a dynamic remodeling of the ECM in the grafts and tissues due to the interaction in the microenvironment. A combination of natural protein and synthetic polymers is preferred to prolong the degradation time and improve the mechanical properties, and one such study was on vascular grafts which are fabricated by coelectrospinning of poly(d,l-lactic acid-*co*-glycolic acid) (PLGA), gelatin, and α-elastin. The results showed that there were no local or systemic toxic effects from grafts when implanted *in vivo* with similar mechanical properties and tissue composition of the scaffolds compared to native vessels.^224^ Biomimicry is the ability of the bioprinted construct to incorporate into the host and provide effective attachment, migration, proliferation, and function to the cells. Various studies have well established that the biomaterials used for generating the bioinks largely influence the attachment of cells. Few surface ligands are also added in the bioinks in order to increase the attachment and proliferation of cells in the bioprinted constructs. The ability to reproduce identical ECM scaffolds using a bioprinting approach would be useful in tissue engineering and regenerative medicine.

### 5.2 Application of Decellularized ECM-Based Bioinks

With the growing demand for the tissue and organ replacement for repair and reconstruction, 3D bioprinted models in the form of patches, organoids, and organ-on-a-chip are developed to meet the increasing demand. The application of these products is attributed to their mimicking of the physical properties and resembles the microenvironment of native tissue. The biofunctionality of the dECM-derived bioinks depends on factors which interact with other bioactive ingredients in the bioink.^225^

Commercial bioprinters are categorically divided into inkjet, laser, and extrusion based bioprinters, and most of the research has applied the extrusion-based bioprinter as a first choice for 3D bioprinting as it provokes less damage to the cells.^226^ Back in 2014, successful bioinks derived from dECM of various tissues including adipose, cartilage, and heart tissues were applied for 3D bioprinting.^81^ Bioink consisting of human dECM was applied in cardiac patches, and hCPCs with GelMA were bioprinted using the extrusion bioprinter model and led to an increase of endogenous cardiomyocytes in the infarct area and improved cardiac function.^13^ Another study was successful in bioprinting with bioink composed of rabbit bone marrow derived stem cells to fabricate functional scaffolds for bone tissue regeneration resulting in an increase in chondrogenesis medium which is a promising way to fabricate *in vitro* cartilage tissue.^12^ A 3D model for human skin was bioprinted using skin-derived dECM. *In vivo* results revealed that an endothelial progenitor cell (EPC)-laden 3D-printed skin patch together with adipose-derived stem cells (ASCs) accelerates wound closure, re-epithelization, and neovascularization as well as blood flow.^227^

The potential idea of obtaining dECM-derived products is meeting a lot of applications and is used commercially. Most of the applications of dECM-derived products include implants for tissue regeneration which also involve a patient-specific decellularization protocol.^228−232^ Clinical studies are comparatively helpful in providing relative and useful information to understand different dECM-derived products as they are specific for the application.^233−235^ There is a vacuole which needs to be covered in the standardization of tissue-specific protocols for decellularization to serve as a comparative study.^15,226,236^Table 2 describes different types of bioink formulated in various studies along with their composition and application.

**Table 2:** 3D Printed Decellularized ECMs and Their Application^a^

Sl. No.	Source of dECM	Bioink Composition	Type of bioprinting	Application	References
1	Heart	GelMA^1^, cardiac dECM^2^	3D bioprinter	Personalized patch	(237)
2	Omenta	Dielectric ink, Omenta dECM	Extrusion-based bioprinter	Provide electrical stimulation for pacing	(238)
3	Skin	GelMA, skin dECM	Extrusion-based bioprinter	Injury repair and regeneration	(239)
4	Kidney	Gelatin, glycerol, kidney dECM, hyaluronic acid 2-hydroxy-1-(4- (hydroxy ethoxy) phenyl)-2-methyl-1-propanone	Cell-based microextrusion printing	Replacement as functional renal tissue construct	(240)
5	Bone	Tempo-oxidized cellulose nanofiber, sodium alginate, cancellous bone dECM	3D bioprinter	Regeneration of cartilage tissue	(241)
6	Heart	GelMA, heart dECM, MTGase^3^	3D bioprinter	*In vitro* model	(242)
7	Meniscus	Meniscus dECM	Stereolithography printing	Meniscus regeneration	(243)
8	Liver	PEGDA^4^ and GelMA	High throughput 3D bioprinter	Drug study on *in vitro* model	(244)
9	Esophagus	Human dermal fibroblasts, human esophageal smooth muscle cells, human bone-marrow-derived mesenchymal stem cells, and human umbilical vein endothelial cells	3D bioprinter	Repair of esophageal defects	(245)
10	Colon	Hyaluronic acid and collagen	Immersion printing technique	*In vitro* tumor model	(246)

a GelMA^1^ - gelatin methacrylamide, dECM^2^ - decellularized extracellular matrix, MTGase^3^ - microbial transglutaminase, PEGDA^4^ - poly(ethylene glycol) diacrylate.

### 5.3 Current Global dECM Market Trend

Decellularized tissue products have attracted considerable attention and market traction in the fields of tissue engineering and regenerative medicine. The global tissue engineering market was estimated at $11.26 billion in 2021 and is expected to reach US$31.23 billion by 2030, growing by 10.46% over the forecast period from 2022 to 2030.^247^ The ECM market is expected to reach $52.72 million in 2028 and $31.49 million in 2021. It is estimated to grow by 7.6% between 2021 and 2028.^248^ Bioink market size is expected to reach $128.63 million by 2021, and total revenue will grow by 20.48% between 2022 and 2029, reaching $571.03 million.^249^ The dECM products have potential benefits such as preserving the components of tissue structure and ECM, promoting tissue regeneration, and repair. The global market for decellularized tissue products has grown steadily with increased research and development efforts, advances in tissue engineering technologies, and increasing demand for regenerative medicine solutions. Table 3 summarizes a few commercially available decellularized tissue-based products.

**Table 3:** Examples of Clinical Products Composed of Decellularized Tissues

Sl. No.	Product name	Tissue source	Company name	Clinical application
1	Dermacell AWM	Human dermis	LifeNet Health	Skin grafts, wound healing
2	CartiMax	Cartilage	MTF Biologics	Cartilage repair, joint reconstruction
3	CorMatrix	Porcine small intestine submucosa	CorMatrix Cardiovascular Sciences	Cardiac tissue repair, pericardial reconstruction
4	SynerGraft	Decellularized heart valve (Allograft)	CryoLife	Heart valve replacement, cardiovascular surgery
5	GraftJacket	Human dermis	Wright Medical	Soft tissue reconstruction, wound healing
6	AlloDerm	Human dermis	LifeCell	Soft tissue reconstruction, breast reconstruction
7	Osteocel	Human bone	NuVasive	Bone grafts, orthopedic surgery
8	VascuCel	Human blood vessels	LeMaitre Vascular	Vascular grafts, vascular surgery
9	MatriStem	Porcine urinary bladder submucosa	Acell	Soft tissue reconstruction, wound healing
10	FlexHD	Human dermis	MTF Biologics	Soft tissue reconstruction, wound healing
11	DuraMatrix	Bovine pericardium	Stryker	Dural repair, neurosurgery
12	CryoPatch	Human pericardium	CryoLife	Cardiac tissue repair, congenital heart surgery
13	AmnioFix	Human amniotic membrane	MiMedx Group	Wound healing, tissue regeneration
14	Actifuse	Porcine bone	Baxter	Bone grafts, orthopedic and spine surgeries
15	InteGra	Porcine peritoneum	LifeNet Health	Abdominal wall reconstruction, hernia repair
16	FlexHD Pliable	Human dermis	MTF Biologics	Soft tissue reconstruction, plastic surgery
17	Matrion	Human placenta	LifeNet Health	Allograft, wound healing
18	AlloPatch HD	Human dermis	ConMed Linvatec	Tendon repair
19	ArthoFlex	Human dermis	LifeNet Health	Soft tissue reconstruction
20	Cortiva	Human dermis	RTI Surgical	Soft tissue reconstruction
21	Axi	Human dermis	Coloplast	Uterus
22	SureDerm	Human dermis	Hans Biomed	Skin transplant
23	Medeor Matrix	Porcine dermis	DSM	Soft tissue reconstruction
24	Permacol Surgical Implant	Porcine dermis	Medtronic	Hernia repair and abdominal wall repair
25	Strattic	Porcine dermis	Strattice Reconstructive Tissue Matrix	Surgical repair, soft tissue reconstruction
26	XenMatri	Porcine dermis	Becton Dickinson	Soft tissue reconstruction
27	Meso BioMatrix	Porcine peritoneum	MTF Biologics	Surgical mesh
28	SurgiMend	Fetal and neonatal bovine dermis	Integra LifeSciences	Hernia repair
29	CopiOs	Bovine pericardium	ZimVie	Dentistry, aesthetic soft tissue repair
30	Perimount	Bovine pericardium	Edwards Lifesciences	Valve replacement
31	Tutopatch	Bovine pericardium	RTI Surgical	Soft tissue reconstruction
32	Matrix patch	Equine pericardium	Autotissue	Valve replacement
33	Chondrofix	Human bone/cartilage	Zimmer Inc.	Knee joint
34	Suspend	Human pascia lata	Coloplast	Urethra
35	Veritas	Bovine pericardium	Baxter	Soft tissue reconstruction

Several key factors have contributed to market growth which include increased prevalence of chronic diseases like cardiovascular diseases, orthopedic conditions, and wound healing complications, which are driving the demand for innovative tissue engineering solutions such as decellularized tissue products. Second, the aging population is more susceptible to degenerative diseases and tissue damage, which increases the need for tissue repair and regeneration options. Third, progress in tissue decellularization technologies and bioengineering methods has improved the quality and effectiveness of tissue decellularized products, making them more clinically viable. Public and private investments in tissue engineering and regenerative medicine research and development activities have stimulated the development of decellularized tissue products. Finally, successful regulatory approvals and clinical trials of decellularized tissue products have increased confidence among medical professionals and patients, resulting in a wider adoption in clinical practice.^250^ The dECM-based bioinks offer several advantages in tissue engineering and regenerative medicine. The most important clinical outcomes of using bioinks based on decellularized tissues include: providing a biomimetic microenvironment for decellularized tissues to retain the architecture of the natural ECM, biochemical signals, and bioactive factors that can provide a biomimetic microenvironment for cells that enhance cell attachment, proliferation, differentiation, and tissue regeneration. The dECM-based bioinks promote a better integration between printed constructs and surrounding host tissues. The preserved components of the ECM facilitate cell migration and tissue remodeling, which improves tissue integration and functional results. The biocompatibility and reduced immune capacity are achieved by eliminating cellular components, hence reducing the risk of immune rejection. This makes dECM-based bioinks more biocompatible and reduces the possibility of immune reactions or side effects when implanted in patients. The customized bioinks based on decellularized tissues can be derived from the patient’s own (autologous) tissue or from allogenous or xenogeneous sources. Autologous bioinks have the advantage of patient-specific treatments for the patient, allowing tissue to be extracted from the patient’s own cells, reducing the risk of rejection of the immune system, and improving overall compatibility. The regeneration and repair of damaged tissue and organs can be used to engineer functional tissues and organs and replace damaged or diseased tissues such as skin, cartilage, bones, blood vessels, etc.^251^ The specific clinical outcomes and benefits of decellularized tissue-based bioinks vary depending on the tissue type used, bioink formulation, printing technology, and target application. Research and clinical studies are being conducted to explore and optimize the clinical potential of decellularized tissue-based bioinks for specific tissue engineering applications. Currently, there are many ECM-based implants around the world that have been successful, such as AlloDerm, Oasis, and Chondro-Gide (Table 3). However, very few dECM-based bioinks are available at this stage of clinical use. Several dECM bioinks have been developed, and several bioprinting companies, such as T&R Biofab and Innoregen, have recently successfully marketed dECM bioinks, including bone deCelluidTM, cartilage deCelluidTM, skin deCelluidTM, and gel4Tissue.^252−254^

## 6.0 Conclusion

Decellularization is an evolving technique for generating organ- or tissue-specific dECM-derived products for various biomedical applications. Generally, decellularization is obtained by a combination of physical, enzymatic, and chemical treatments of the *in vitro* organs. With the rise in the application of tissue engineering, there is an increase in the demand for biomaterials such as decellularized tissues. The choice of method for decellularization is very crucial, as the source is either allogeneic or xenogeneic. Further standardization of protocols for evaluating decellularization is required for obtaining efficient results. Many studies and research have proven that the decellularization of various tissues is possible, but at the same time all of them are at a small scale and applied in laboratories. Due to the lack of standardized protocols for decellularization, a major challenge still remains in the production of large-scale decellularized materials for commercialization that will require more time. Research involving optimization will lead to the development in applications for regenerative medicine and improvement in healthcare. The dECM-based products have been applied for various studies such as transplantation, drug screening, repair, and regeneration. A wide range of decellularized products have come to approval for application on patients including human dermis (Alloderms, LifeCell, Corp.), porcine SIS (SurgiSISs, Cook Biotech, Inc.; Restores, DePuy Orthopedics, Inc.), porcine urinary bladder (ACell, Inc.), and porcine heart valves (Synergrafts, CryoLife, Inc.). There is a growing list of bioinks for preparing different biological scaffolds for clinical applications.
